# A Critical Reanalysis of Uncontrollable Washboarding Phenomenon in Metal Band Sawing

**DOI:** 10.3390/ma13204472

**Published:** 2020-10-09

**Authors:** Kazimierz A. Orlowski, Michal Dobrzynski, Grzegorz Gajowiec, Marcin Lackowski, Tomasz Ochrymiuk

**Affiliations:** 1 Department of Manufacturing and Production Engineering, Faculty of Mechanical Engineering, Gdansk University of Technology, Gabriela Narutowicza 11/12, 80-233 Gdańsk, Poland; kazimierz.orlowski@pg.edu.pl (K.A.O.); michal.dobrzynski@pg.edu.pl (M.D.); 2 Department of Materials Engineering and Bonding, Faculty of Mechanical Engineering, Gdansk University of Technology, Gabriela Narutowicza 11/12, 80-233 Gdańsk, Poland; grzgajow@pg.edu.pl; 3 Institute of Fluid-Flow Machinery Polish Academy of Sciences, 80-233 Gdańsk, Poland; mala@imp.gda.pl

**Keywords:** metal band sawing, washboarding phenomenon, empirical test, 3D cut surface examination, waviness profiles analyses

## Abstract

The article analyzes the cutting process of hard bars. Investigations conducted in industrial conditions demonstrated the presence of surface errors in the machined workpieces in the form of washboard patterns. The purpose of this study was to analyze the results of cutting on band sawing machines with different band saw blades. The cutting processes were conducted on three different horizontal band sawing machine types. Analyzed material was an alloy steel Ø40 mm rod with a hardened surface covered with a thin layer of chromium. The hardness of the outer layer was 547 HV with a core hardness of 180 HV. The surface topography measurements of the processed workpieces were carried out with the 3D Optical Profiler, which supplied information on the irregularities of the processed material texture. In each of the analyzed cases, a corrugated surface was obtained after sawing, which is the effect of the occurrence of the washboarding phenomenon, despite the fact that the teeth of each band saw had variable pitches. The washboarding phenomenon when cutting rods with hard surfaces is caused by the phenomenon of wave regeneration. Despite the use of variable pitch saw blades, the cutting process results in rippling of the sawn surface, which is caused by the high hardness of the outer layer of the workpiece, as well as by the type of tool with spring setting of teeth.

## 1. Introduction

Round materials and profiles can be cut on circular, frame, and band sawing machines. The latter group is widely used because of the possibility of process automation, small kerf losses, straightness of the kerf, good surface quality, as well as long saw blade life [1]. Cutting with band saw blades is characterized by the fact that the thicknesses of the cut layers are small, the nature of the cutting edges work is intermittent [2], the number of cutting edges in contact with the workpiece can change, and the saw blade itself is an object of low own stiffness [3,4].

Although in many cases cutting of rods is done on CNC-controlled band saws, the cutting performance is worse than that achieved by modern circular saws. Due to the quality of the surface obtained after the band sawing operation, at least two turning operations are always required when machining the face surfaces. The first treatment is a coarse treatment for the initial achievement of the required dimension and leveling of potential irregularities (perpendicularity error), and the next is the finishing operation for obtaining the final dimension and the required surface roughness. Conversely, after cutting on a circular sawing machine, the initial turning operation is omitted and only the finishing operation is performed [5].

As a result of the fact that cutting is the first technological operation, the effects of which are often removed during subsequent operations, there are not many scientific works in this area. A common case of errors when cutting with band saws is the occurrence of a characteristic wave on the cut surface. In recent years, a number of papers analyzed the problems of loss of stability in the cutting process of band saws where the authors

- Used signals of sound pressure, vibrations and cutting forces for the proprietary method of detecting chatter vibrations, which in this case were independent of the workpiece geometry and cutting parameters [6,7];
- Developed a model in which the free length of the saw blade, cutting speed, and feed speed were taken into account to determine stability regions, which avoid the presence of chatter vibrations when cutting [8]. Two strategies have been proposed to avoid this type of phenomenon, the first of which is to reduce and the second to increase the cutting speed to find working parameters outside the area where there would be a risk of loss of stability [8,9].

**Figure 1:**
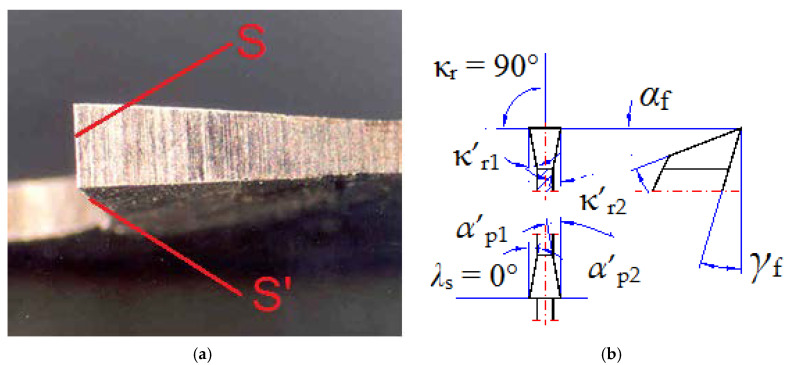
Band saw tooth general view from the top (**a**) and its geometry in the tool-in-hand system (**b**), where S—main cutting edge, S’—minor cutting edge, *α*_f_ the tool side flank, *α*’_p_ the tool back flank, κ_r_ tool cutting edge angle, κ’_r_ minor tool cutting edge angle, *γ*_f_ the tool side rake, and *λ*_s_ the cutting edge inclination angle.

It should be emphasized that chatter vibrations are the result of a number of phenomena during the cutting process occurring in the cutting zone on the main cutting edge [10,11]. On the other hand, when cutting with saws, three cutting edges are involved in cutting, for the simplest geometry, the main edge, and two minor cutting edges (Figure 1). The position of the minor cutting edges S’ is defined by the minor tool cutting edge angles κ’_r_ (Figure 1b).

Phenomena occurring on the minor cutting edges, caused by back forces (perpendicular to the saw blade) are responsible for the formation of waviness of the cut surface [12,13]. Hereafter, to distinguish from chatter vibrations, the phenomenon of surface rippling when cutting with saws due to loss of stability will be called the washboarding, as it was done by Okai [14] and Orlowski and Wasielewski [15]. Ni et al. [3] developed and experimentally verified the dynamic model of cutting forces when cutting on band saws. The problems of wear of teeth of saw blades while cutting bearing steel were described by Sarwar et al. [1]. Boland et al. [16] provided an overview of the developments in diamond composite materials with particular emphasis on microstructural characterization. Those investigations greatly help in the design of innovative diamond tools for cutting, drilling, and sawing processes. The 3D topography measurements allow one to perform a comprehensive quantitative evaluation of a surface after cutting. The evaluation of surface texture formed during turning of CoCr alloys fabricated by casting and SLM processes was presented by Krawczyk et al. [17]. The influence of cutting parameters (speed, feed rate, and depth) during dry turning on the areal surface roughness of AZ31B Magnesium Alloy was thoroughly investigated by Gao et al. [18].

When cutting hard bars on band sawing machines in industrial conditions, geometric errors generated in the sawn surfaces by cutting take the form of washboard patterns. The present study aims to analyze in-depth the results of cutting operations on band sawing machines with different band saw blades.

## 2. Materials and Methods

### 2.1. Materials

**Figure 2:**
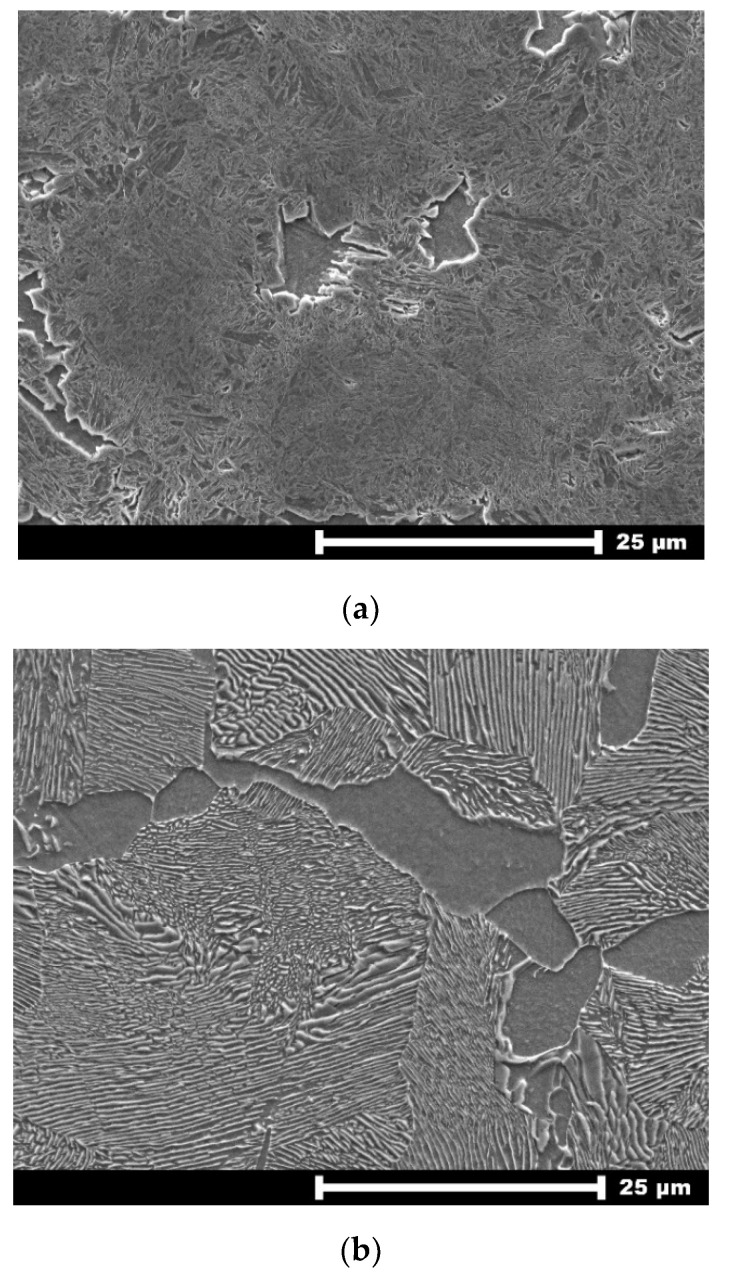
Rod metallographic structures: martensitic-ferritic outer layer (**a**) and pearlitic-ferritic core (**b**), obtained by SEM electron microscope (magnification 2000×).

In the analyzed cases, the cut material was a rod with a diameter of Ø40 mm from alloy steel (grade of steel 66Mn4 (1.1260)) with a hardened surface. Moreover, its surface was covered with a thin layer of chromium. The hardness of the outer layer was 547 HV (about 52 HRC), while core hardness was 180 HV. HV hardness was determined on the Vickers-Brinell hardness tester HPO 250 (Hegewald & Peschke Meß- und Prüftechnik GmbH, Nossen Germany). The structure of the hardened layer (547 HV) of the workpiece (with a thickness of about 2 mm) was mainly made by martensite with a small amount of ferrite, as shown in Figure 2a. On the other hand, Figure 2b shows the structure of the rod core (180 HV), in which pearlite and ferrite are observed.

### 2.2. Types of Cutting Machines and Tools

The cutting process case #1 was conducted on the horizontal band sawing machine type JB250 (unknown producer) with a tool of technical data: bimetal bandsaw, back to tip tooth height B = 27 mm, blade thickness s = 0.9 mm, variable pitch (P) 4/6 teeth per inch (TPI), teeth set pattern: straight-right-left sequence; tooth tip material: high speed steel M71-C (1000 HV, according to American Iron and Steel Institute (AISI)). Cutting parameters: cutting speed v_c_ = 34 m·min^−1^ and feed speed 6.7 mm·min^−1^ (feed per tooth f_z_ ≈ 1 µm).

In the case #2, the cutting process was carried out on the horizontal band sawing machine type HBP220A (producer Behringer, D, Kirchardt, Germany) with a tool of technical data: bimetal bandsaw, back to tip tooth height B = 34 mm, blade thickness s = 1.1 mm, variable pitch (P) 3/4 TPI, teeth set pattern: straight-right-left sequence; tooth tip material high: speed steel M42 (950 HV, according to AISI). Cutting parameters: cutting speed v_c_ = 27 m·min^−1^ and feed speed 1.1 mm·min^−1^ (feed per tooth f_z_ ≈ 0.3 µm).

In the case #3, the cutting process was realized on the horizontal band sawing machine type HBP413A (producer Behringer, D) with a tool of technical data: bimetal bandsaw, back to tip tooth height B = 41 mm, blade thickness s = 1.3 mm, variable pitch (P) 2/3 TPI, teeth set pattern: straight-right-left sequence; tooth tip material high: speed steel M42 (950 HV, according to AISI). Cutting parameters were cutting speed v_c_ = 23 m·min^−1^ and feed speed 3 mm·min^−1^ (feed per tooth f_z_ ≈ 1.3 µm).

It ought to be emphasized that cutting parameters were taken from Perschmann handbook [19] and suggested by the experienced engineers from the plant, where tests were conducted.

Basic technical data of bandsaws used in experiments are presented in Table 1.

### 2.3. Cut Surface Examination

The surface topography measurements of the processed workpieces were carried out with the 3D Optical Profiler S neox (Sensofar 2019, Sensofar Metrology, Terrassa, Spain) [20] with objective 5× EPI of Nikon. The system was controlled by SensoSCAN 6.6 software (v.6.6, Sensofar Metrology, Terrassa, Spain), and the basic details of the measurement were set as follows:

- Topography: 8759 × 8770 px (Sample K#1), 16,377 × 16,662 px (Sample K#2) and 7846 × 8332 px (Sample K#3);
- Pixel size: 5.2 µm/pixel for Sample K#1 and K#3, 2.6 µm/pixel for Sample K#2;
- Axis Z-Scan step of 12 μm;
- Threshold 3%;
- Algorithm: Confocal Fusion.

Surface analyses were conducted with MountainsMap 7.1 software package (2019, Digital Surf made available Optotom,) Warsaw, Poland, Besançon, France. In order to analyze texture, the general slope of a sample was removed using a levelling process. Therefore, the ‘’Level’’ Operator was applied, in accordance with ISO 25178 [21], based on the least-squares form fitting levelling, using an LS-plane. Additionally, the median-type spatial filtering of 3 × 3 and 9 × 9 size for measured samples were used. The purpose of these filters was to reduce the noise in an image and the microroughness on analyzed surfaces.

**Figure 3:**
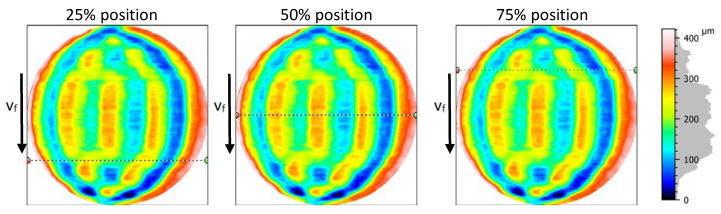
Positions of the control paths defined for sample K#3 for extracting W-profiles based on waviness surfaces, where arrow v_f_ is a direction feeding, and v_f_ is feed speed.

On the S-F surfaces (surface derived from the primary surface by removing the form, in accordance with ISO 25178-2 [22]) three control paths lying on parallel lines with a constant spacing of 25% of the ROI size were defined for extracting W-profiles from the S-F surfaces (Figure 3).

## 3. Results and Discussion

**Figure 4:**
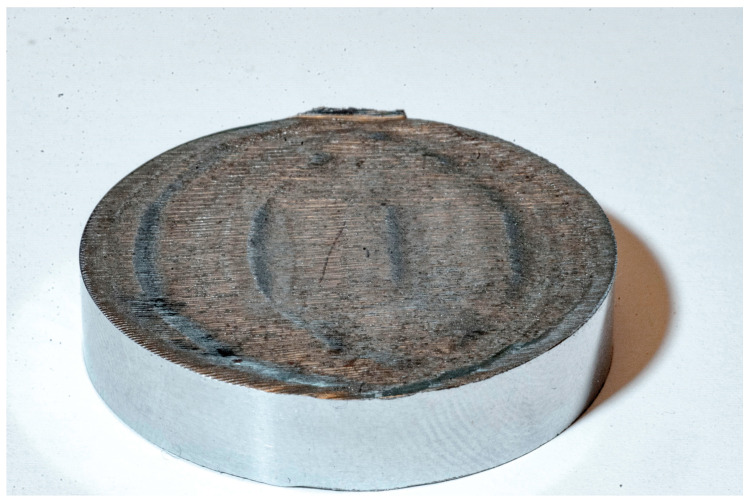
General view of the sample #1 with the washboard pattern on the sawn surface.

In each of the analyzed cases, a corrugated surface was obtained after sawing (Figure 4), which is the effect of the occurrence of the washboarding phenomenon, despite the fact that the teeth of each band saw had variable pitches (#1—4/6 TPI, #2—3/4 TPI and #3—2/3 TPI), which should prevent instability in the machining process according to the theory [11].

Kimura et al. [12] have shown that under certain conditions, the use of variable pitches of a band saw teeth does not produce the desired results. Moreover, changes of impact frequencies on the sash gang saw were in the range 0 to several hundred Hz in one cycle; saw blades with spring setting of teeth, which had minor-cutting-edge feathers after grinding, did not prevent this type of waviness (presence of washboarding pattern) [15].

**Figure 5:**
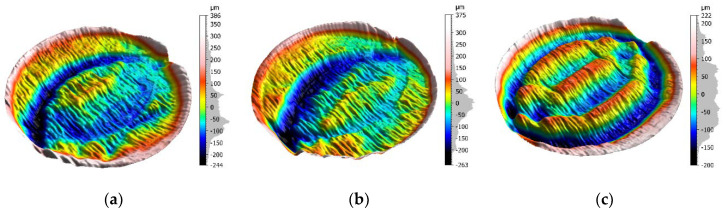
3D waviness surface under investigation of the samples #1 (**a**), #2(**b**), and #3 (**c**).

In Figure 5, exemplary 3D waviness surfaces of the samples #1, #2 and #3 are presented. All S-F surfaces of samples were obtained by applying a Gaussian filter, in accordance with ISO 16610-61 [23]. The choice of the nesting index (cut-off) of 0.8 mm was connected with obtaining a surface texture with visible reliefs of the workpiece surface after sawing.

The main height parameters of the S-F sample surfaces: Sq—root mean square roughness, Sp—maximum peak height, Sv—maximum valley depth, and Sz—maximum height of the surface, in accordance with ISO 25178 [24], are presented in Table 2. 

The height parameters of the S-F surfaces (Table 1) point out that the maximum height between the edge of the sample and the maximum depth of the furrows is much larger for samples #1 and #2. In the case of sample #3, such value reaches 422 µm, and it is 200 µm smaller than for other samples. Sp and Sv parameters of the sample #3 indicate regular spacing of hills and valleys.

**Figure 6:**
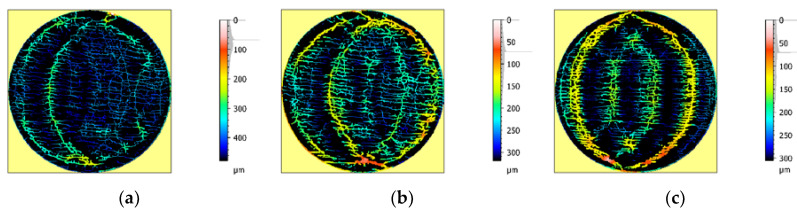
View of the furrows of the samples #1 (**a**), #2 (**b**), and #3 (**c**).

Further study supplied information about the maximum and the mean depth of the furrows (Figure 6).

Data on furrow depth and density gathered from the examined surfaces are presented in Table 3. The obtained mean values oscillated around 100 µm, the maximum depth of samples of furrows for sample #3 was about 200 µm lower than for sample #1. In all cases, the density of the furrows, defined as a cumulative length per area unit (in 1/cm), were at the similar level.

Table 3 lists the waviness parameters extracted from the W-profiles corresponding to the three parallel control paths defined for each specimen; the cut-off value of profiles (set as 0.8 mm (ISO 4287 [24])) was collected in Table 4. Parameters in Table 4 are as follows: Wp—waviness maximum profile peak height, Wv—waviness maximum profile valley depth, Wz—maximum height of the waviness profile, and Wq—waviness root mean square deviation.

**Figure 7:**
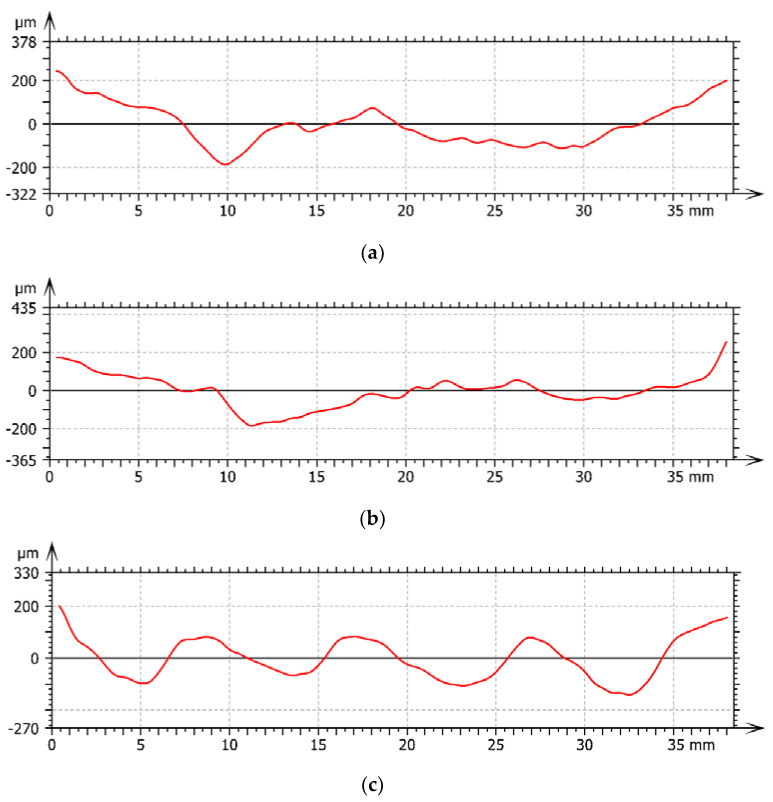
Exemplary W—profile at 50% position of the samples #1 (**a**), #2 (**b**), and #3 (**c**).

An exemplary W-profile at 50% position of the sample #3 is presented in Figure 7. According to the values of waviness parameters and profile information given in Table 3, the middle of the machined samples #1 and #2 was distinctly above the edge, 2–3 µm wide, and with deep furrows. In sample #3, all of the furrows after machining process were well visible and their height was similar level to regular spacing of hills and valleys.

The use of variable teeth pitches of teeth in all experiments did not result in machined surfaces without waviness errors. The average values of the excitation frequencies were for the cases #1 f_a_ = 110 Hz, #2 f_a_ = 62 Hz, and #3 f_a_ = 37.7 Hz, respectively.

The phenomenon of the occurrence of the corrugated surfaces after sawing in this case could be due to the behavior of the saw blade with spring setting teeth caused by the high hardness of the outer layer of the sawn rod (547 HV). For spring setting teeth, the saw blade during entrance into cut material is twisted by the lateral forces (back forces). Under specific cutting conditions, while the washboard pattern is formed, every successive cutting edge regenerates a wave performed by the previous one. Nevertheless, this new wave is part of a distance velocity lag in the direction of primary motion. The result of this wave regeneration on the sawn surface is visible as a washboard pattern, which can be described by parameters of waviness. The wave regeneration in the examined cases is not a time function but a function of the saw blade position and simultaneous effect of forced excitations of the formed wave upon the saw blade lateral displacements. This phenomenon was described in detail by Orlowski and Wasielewski [15], who analyzed process of sawing beech and pine wood on the sash gang saw PRW15-M. In the sawing process, they used saw blades of poor quality of teeth cutting edges after grinding (minor cutting edges with feathers). It should be emphasized that waviness parameters for those species were almost equal.

In the described cases we are dealing with, the opposite phenomenon—the role of the object forcing the behavior of the saw blade—is played by the hard outer layer of the workpiece, not by the poor quality of the tool blade.

## 4. Conclusions

Despite the use of variable pitch saw blades in the process of cutting on different band saws, there may be a washboarding phenomenon, resulting in rippling of the sawn surface, which is caused by the high hardness of the outer layer of the workpiece, as well as by the type of tool with spring setting of teeth.

The conducted surface topography measurements of the processed workpieces, which were carried out with the 3D Optical Profiler S neox, revealed that waviness height parameters were at a similar level. Nevertheless, the small reduction in Wz maximum height of the waviness profile is observed with an increase of teeth pitches.

As when shaping the washboard pattern while sawing wood with poor quality of teeth cutting edges, the washbording phenomenon when cutting rods with hard surfaces with bandsaws, which have spring setting teeth, is caused by the phenomenon of wave regeneration.

Based on the study, the performed application of bandsaws with spring setting teeth is strongly discouraged for cutting-off hard-to-machine materials. If this type of bandsaw is used, in the next step another technological operation, e.g., turning, is needed. On the other hand, with such a difficult technological task, cutting-off wire electro discharging machining (WEDM) could be recommended.

## Figures and Tables

**Table 1:** Basic technical data of bandsaws.

Case	B [mm]	s [mm]	P [TPI]	Set Pattern
#1	27	0.9	4/6	straight-right-center
#2	34	1.1	3/4	straight-right-center
#3	41	1.3	2/3	straight-right-center

Legend: TPI—number of teeth per inch; pattern: repeatable teeth grouping; straight-teeth not bent, right—teeth bent in the right side, left—teeth bent in the left side.

**Table 2:** Main height parameters of the S-F examined sample surfaces.

-	#1	#2	#3
Sq [μm]	109.97	96.62	80.29
Sp [μm]	386.34	375.35	222.10
Sv [μm]	244.25	263.39	200.32
Sz [μm]	631.59	638.73	422.42

**Table 3:** Parameters of furrows contained in examined sawn samples.

	#1	#2	#3
Max. depth of furrows [µm]	474.40	314.80	290.86
Mean depth of furrows [µm]	103.13	107.27	98.05
Mean density of furrows [1/cm]	13.92	13.38	13.01

**Table 4:** Waviness parameters of the profiles extracted in selected positions of samples.

-	#1			#2			#3		
	Position			Position			Position		
	25%	50%	75%	25%	50%	75%	25%	50%	75%
Wp [µm]	267.83	247.81	250.39	359.34	260.33	262.72	183.97	204.75	160.60
Wv [µm]	185.38	182.00	127.49	166.92	181.10	142.38	134.36	137.18	117.21
Wz [µm]	453.21	429.81	377.88	526.26	441.43	405.10	318.33	341.93	277.81
Wq [µm]	119.32	93.15	91.50	97.08	82.11	76.60	75.04	77.10	63.56
